# Supplementary material for: Haplotype-Resolved, Chromosome-Level Assembly of White Clover (Trifolium repens L., Fabaceae)
Source: Genome Biol Evol. 2023 Aug 5;15(8):evad146. doi: 10.1093/gbe/evad146 (PMC10433932; doi:10.1093/gbe/evad146)
Supplement: evad146_Supplementary_Data [file evad146_supplementary_data.docx]

**Supplemental materials**

**
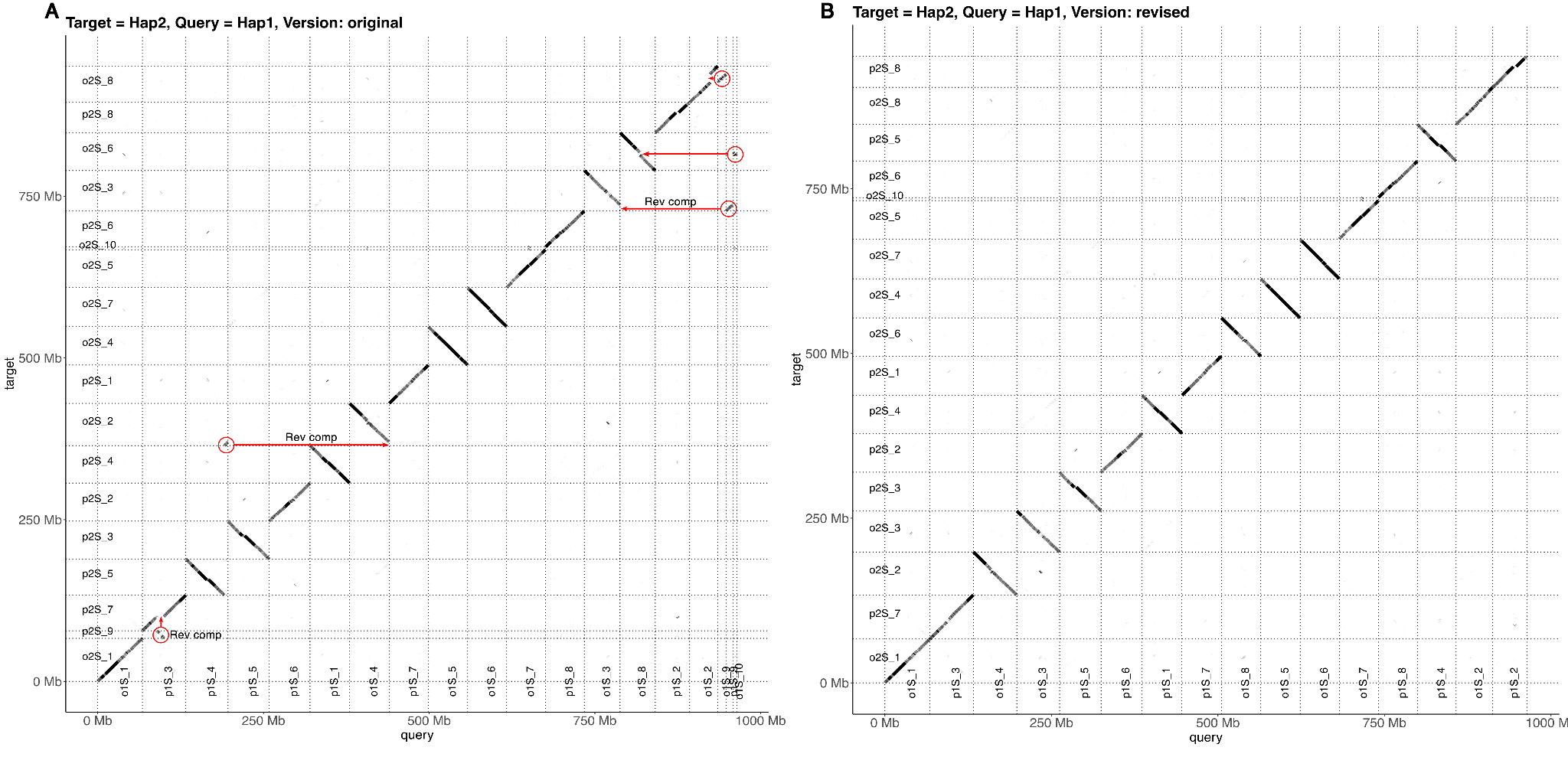
**

**Figure S1:** Original Dovetail haplotypes (A) and revised haplotypes (B) following manual fixes implemented in BioPython. Red circles surround fragments (often entire unplaced scaffolds) that map to gaps or telomeric regions of assembled chromosomes. Red arrows indicate where fragments were placed, and whether they were reverse-complemented before placement.

**
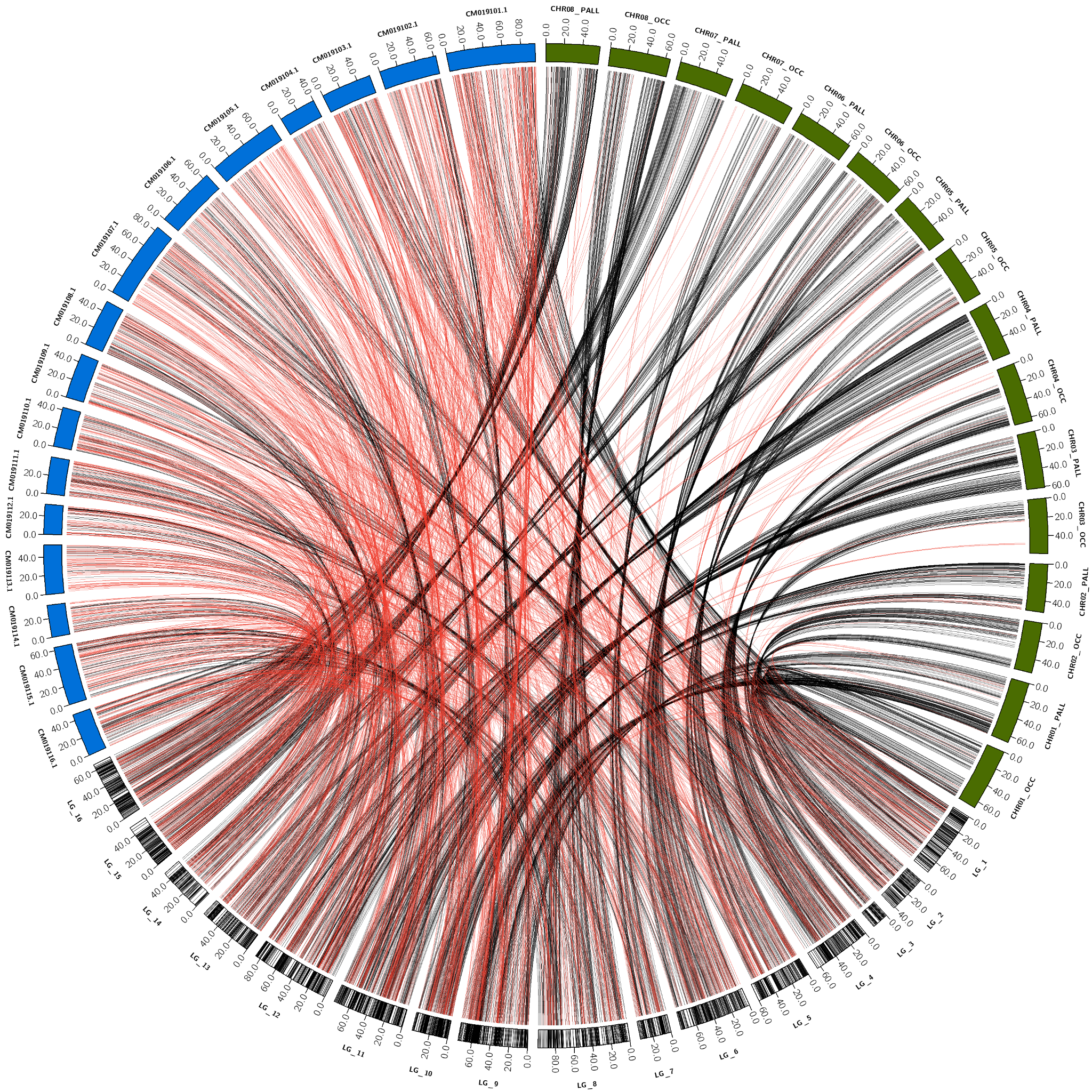
**

**Figure S2:** Linkage map from the “SG” mapping population ([23], bottom, converted to physical positions in Mbp) with markers (vertical black lines in ideogram) connected to their physical positions in both the previous reference assembly (blue) and the current haploid assembly (green). Lines connecting markers to their physical position are colored red if they map to the wrong chromosome based on the linkage data, or black if they map to the correct chromosome. 94.6% (N = 2,049) of the 2,165 filtered linkage markers mapped to the correct chromosome in our new assembly, compared to 40.0% (N = 867) in the previous assembly.

**Supplementary tables**

**Table S1:** Genome BUSCO scores for all assemblies and Protein BUSCO scores for haploid mapping assembly against the embryophyta and fabales lineage datasets.

|  | *Genome-mode* | | *Protein-mode* | |
| --- | --- | --- | --- | --- |
|  | **Embryophyta_odb10 (N = 1,614)** | **Fabales_odb10 (N = 5,366)** | **Embryophyta_odb10 (N = 1,614)** | **Fabales_odb10 (N = 5,366)** |
|  | Haploid | | Haploid | |
| **Complete BUSCOs** | 99.6% (1,607) | 99.5% (5,340) | 99.6% (1,607) | 99.3% (5,328) |
| **Complete & single copy** | 5.3% (85) | 4.1% (221) | 3.9% (63) | 4.9% (264) |
| **Complete & duplicated** | 94.3% (1,522) | 95.4% (5,119) | 95.7% (1,544) | 94.4% (5,063) |
| **Fragmented** | 0.2% (3) | 0.1% (3) | 0.1% (2) | 0.1% (7) |
| **Missing** | 0.2% (4) | 0.4% (23) | 0.3% (5) | 0.6% (31) |
|  | Haplotype 1 | |  |  |
| **Complete BUSCOs** | 99.6% (1,608) | 99.5% (5,341) | – | – |
| **Complete & single copy** | 5.9% (95) | 4.1% (221) | – | – |
| **Complete & duplicated** | 93.7% (1,513) | 95.4% (5,120) | – | – |
| **Fragmented** | 0.2% (3) | 0.1% (3) | – | – |
| **Missing** | 0.2% (3) | 0.4% (22) | – | – |
|  | Haplotype 2 | |  |  |
| **Complete BUSCOs** | 99.5% (1,607) | 99.5% (5,339) | – | – |
| **Complete & single copy** | 5.6% (91) | 4.2% (225) | – | – |
| **Complete & duplicated** | 93.9% (1,516) | 95.3% (5,114) | – | – |
| **Fragmented** | 0.2% (4) | 0.1% (3) | – | – |
| **Missing** | 0.3% (3) | 0.4% (24) | – | – |

**Table S2:** Type, number, length, and proportion of repeat elements in the *T. repens* haploid mapping assembly based on Repeat Modeler and Repeat Masker analysis.

| **Element type** | **# elements** | **Length (Mbp)** | **%** |
| --- | --- | --- | --- |
| **Retroelements** | 442,794 | 289.91 | 29.94 |
| **SINEs:** | 8,244 | 1.12 | 0.12 |
| **Penelope** | 12 | 0.00 | 0.00 |
| **LINEs:** | 58,078 | 25.84 | 2.67 |
| **CRE/SCLACS** | 6,716 | 1.46 | 0.15 |
| **L2/CR1/Rex** | 0 | 0.00 | 0.00 |
| **R1/LOA/Jockey** | 0 | 0.00 | 0.00 |
| **R2/R4/NeSL** | 0 | 0.00 | 0.00 |
| **RTE/Bov-B** | 3,328 | 0.61 | 0.06 |
| **L1/CIN4** | 47,990 | 23.77 | 2.45 |
| **LTR Elements:** | 376,472 | 262.94 | 27.16 |
| **BEL/Pao** | 0 | 0.00 | 0.00 |
| **Ty1/Copia** | 171,762 | 130.66 | 13.49 |
| **Gypsy/DIRS1** | 95,923 | 93.76 | 9.68 |
| **Retroviral** | 788 | 0.30 | 0.03 |
| **DNA Transposons** | 89,677 | 17.43 | 1.80 |
| **hobo-activator** | 21,067 | 4.06 | 0.42 |
| **Tc1-IS630-Pogo** | 3,723 | 0.45 | 0.05 |
| **En-Spm** | 0 | 0.00 | 0.00 |
| **MULE-MuDR** | 27,637 | 6.18 | 0.64 |
| **PiggyBac** | 0 | 0.00 | 0.00 |
| **Tourist/Harbinger** | 10,723 | 2.14 | 0.55 |
| **Other (e.g., Mirage)** | 3 | 0.00 | 0.00 |
| **Rolling-circles** | 17,634 | 5.43 | 0.56 |
| **Unclassified** | 967,801 | 261.89 | 27.05 |
| **Total interspersed** | – | 569.23 | 58.79 |
| **Small RNA** | 3,868 | 1.33 | 0.14 |
| **Satellites** | 4,895 | 0.83 | 0.09 |
| **Simple repeats** | 1,270 | 0.06 | 0.01 |
| **Low complexity** | 0.00 | 0.00 | 0.00 |

**Table S3:** RNAseq accessions used for genome annotation using BRAKER.

| **Accession** | **Tissue** | **# Bases (Gbp)** | **Selection** | **Reference** |
| --- | --- | --- | --- | --- |
| SRR12578192 | Root | 6.9 | oligot-dT | [47] |
| SRR12578220 | Root | 7.8 | oligot-dT | [47] |
| SRR12578237 | Root | 7.6 | oligot-dT | [47] |
| SRR12578285 | Root | 10.2 | oligot-dT | [47] |
| SRR12578290 | Root | 8.2 | oligot-dT | [47] |
| SRR8691037 | Root | 19.5 | cDNA | [2] |
| SRR8691038 | Stolon | 17 | cDNA | [2] |
| SRR8691039 | Leaf | 17.7 | cDNA | [2] |
| SRR8691040 | Flowers | 18.7 | cDNA | [2] |
| SRR21383273 | Root | 5 | Random | [73] |
| SRR21383275 | Root | 4.8 | Random | [73] |
| SRR21383276 | Root parasitized by Cuscuta | 4.6 | Random | [73] |
| SRR21383278 | Root parasitized by Cuscuta | 4.7 | Random | [73] |
| SRR20706441 | Root | 5 | Random | [73] |
| SRR20706444 | Root | 4.8 | Random | [73] |
| SRR5814845 | Flowers | 5.4 | cDNA | [74] |
| SRR5814846 | Flowers | 6.6 | cDNA | [74] |
| SRR5814847 | Flowers | 6.3 | cDNA | [74] |
| SRR5814848 | Flowers | 7.3 | cDNA | [74] |
| SRR5814849 | Flowers | 6.2 | cDNA | [74] |
| SRR5814850 | Flowers | 6.1 | cDNA | [74] |
